# Potential Therapeutic Effects of Curcumin on Glycemic and Lipid Profile in Uncomplicated Type 2 Diabetes—A Meta-Analysis of Randomized Controlled Trial

**DOI:** 10.3390/nu13020404

**Published:** 2021-01-27

**Authors:** Emma Altobelli, Paolo Matteo Angeletti, Ciro Marziliano, Marianna Mastrodomenico, Anna Rita Giuliani, Reimondo Petrocelli

**Affiliations:** 1 Department of Life, Public Health and Environmental Sciences, University of L’Aquila, 67100 L’Aquila, Italy; paolomatteoangeletti@gmail.com (P.M.A.); c.marziliano@univaq.it (C.M.); mariannamastrodomenico@gmail.com (M.M.); annarita.giuliani@univaq.it (A.R.G.); 2 Epidemiology and Biostatistics Unit, Local Health Unit, 64100 Teramo, Italy; 3 Rianimazione e TIPO Cardiochirurgica, Ospedale G. Mazzini, Local Health Unit, 64100 Teramo, Italy; 4 Public Health Unit, ASREM, 86100 Campobasso, Italy; reimondo.petrocelli@asrem.org

**Keywords:** curcuma, turmeric, type 2 diabetes, dyslipidemia, meta-analysis, randomized control trial

## Abstract

Diabetes mellitus is an important issue for public health, and it is growing in the world. In recent years, there has been a growing research interest on efficacy evidence of the curcumin use in the regulation of glycemia and lipidaemia. The molecular structure of curcumins allows to intercept reactive oxygen species (ROI) that are particularly harmful in chronic inflammation and tumorigenesis models. The aim of our study performed a systematic review and meta-analysis to evaluate the effect of curcumin on glycemic and lipid profile in subjects with uncomplicated type 2 diabetes. The papers included in the meta-analysis were sought in the MEDLINE, EMBASE, Scopus, Clinicaltrials.gov, Web of Science, and Cochrane Library databases as of October 2020. The sizes were pooled across studies in order to obtain an overall effect size. A random effects model was used to account for different sources of variation among studies. Cohen’s *d*, with 95% confidence interval (CI) was used as a measure of the effect size. Heterogeneity was assessed while using Q statistics. The ANOVA-Q test was used to value the differences among groups. Publication bias was analyzed and represented by a funnel plot. Curcumin treatment does not show a statistically significant reduction between treated and untreated patients. On the other hand, glycosylated hemoglobin, homeostasis model assessment (HOMA), and low-density lipoprotein (LDL) showed a statistically significant reduction in subjects that were treated with curcumin, respectively (*p* = 0.008, *p* < 0.001, *p* = 0.021). When considering HBA1c, the meta-regressions only showed statistical significance for gender (*p* = 0.034). Our meta-analysis seems to confirm the benefits on glucose metabolism, with results that appear to be more solid than those of lipid metabolism. However, further studies are needed in order to test the efficacy and safety of curcumin in uncomplicated type 2 diabetes.

## 1. Introduction

Type 2 diabetes (T2DM) is an important issue for public health, and it is growing in the world; in fact, according to the World Health Organization (WHO) report in 2016, 422-million people are diagnosed with diabetes [1]. In Europe, 50% of Countries show T2DM prevalence rates in the range of 8–9% [2]. Bommer et al. have demonstrated that the global costs of T2DM and its consequences are large, and they will substantially increase by 2030 [3].

Lifestyle risk factors that are related to diabetes, as obesity and overweight are two major risk factors. The treatment of T2DM includes the use of anti-diabetic drugs and prevention based on lifestyle habits. In recent years, there has been growing research interest on the efficacy evidence of curcumin use in the regulation of glycemia and lipidaemia [4].

Curcumin is the main bioactive component that is extracted from the rhizome of Curcuma Longa. It is a product used since ancient times, in cuisine, as in traditional medicine [5]. The properties that are attributed to curcumin are remarkable: in fact, it has an antioxidant and anti-inflammatory effect [6].

Its molecular structure makes it possible to intercept reactive oxygen species (ROI) that are particularly harmful in chronic inflammation and tumorigenesis models [5].

Curcumin can have a therapeutic effect on some chronic diseases, such as rheumatoid arthritis, coronary artery disease, atherosclerosis, T2DM, and obesity [7].

Ramirez-Bosca et al. showed that daily treatment with curcumin can decrease the low-density lipoprotein (LDL) in healthy subjects [8]. In addition, Mohammadi et al. [9] demonstrated that one-month oral administration of curcumin (1 gram/day) could reduce the triglycerides concentrations in obese subjects. Rahimi et al., in a randomized trial, highlighted that curcumin reduces HbA1c during three months of therapy [10].

In order to evaluate its effectiveness, some studies have been conducted on the effects of curcumin on glycemic and lipid control in subjects with uncomplicated T2DM [10,11,12,13,14,15,16].

In this systematic review and meta-analysis, we aim to evaluate the effect of curcumin on glycemic and lipid profile in subjects with uncomplicated T2DM.

## 2. Materials and Methods

The papers that were included in the meta-analysis were sought in the MEDLINE, EMBASE, Scopus, Clinicaltrials.gov, Web of Science, and Cochrane Library databases as of October 2020. The search terms used were: curcuma or curcumins or turmeric AND type 2 diabetes or diabetes; (“Diabetes Mellitus, Type 2” [Mesh]) AND (“Curcuma” [Mesh]) and applied the following filters: humans, published articles from 2000 to 2020, and clinical trials.

**Figure 1:**
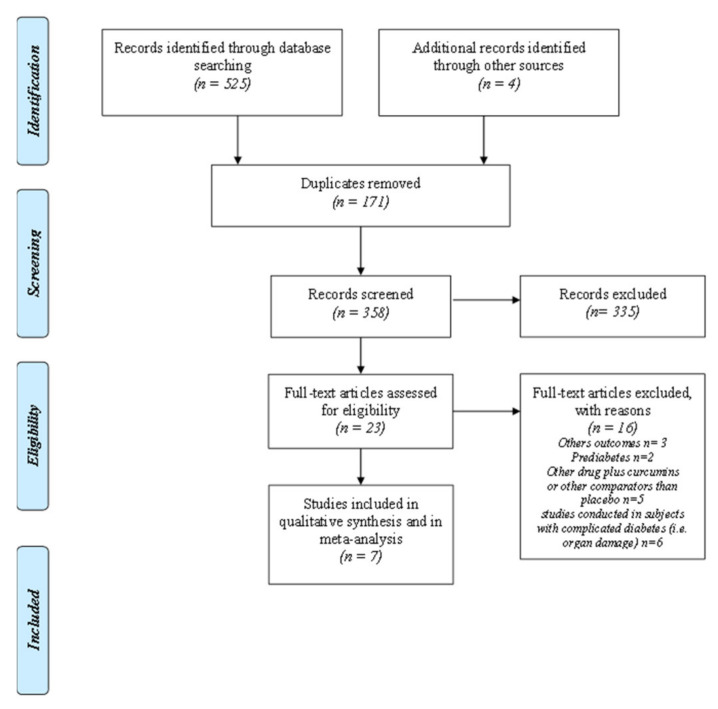
Prisma Flow-chart.

The papers were selected while using the Preferred Reporting Items for Systematic Reviews and Meta-Analyses (PRISMA) flowchart (Figure 1) and the PRISMA checklist (Table S1) [17]. A manual search of possible references of interest was also performed. Only studies that were published in English were considered. The papers were selected by two independent reviewers (P.M.A. and C.M); a methodologist (E.A.) resolved any disagreements. Bias was assessed using the Cochrane Collaboration tool for assessing the risk of bias (Table S2) [18].

## 3. Statistical Analysis

The sizes were pooled across studies in order to obtain an overall effect size. A random effects model was used to account for the different sources of variation among studies [19]. Cohen’s d, with 95% confidence interval (CI) and *p*-value, was used as a measure of effect size [20]. Heterogeneity was assessed using Q statistics, I^2^, Tau, and Tau2. The stability of study findings was checked with moderator analysis. A subgroups analysis was also performed while considering the country of primary studies, because four of seven studies were conducted in Iran. The ANOVA-Q test was used to value the differences among groups. Publication bias was analyzed and represented by a funnel plot; funnel plot symmetry was assessed with Egger’s test [21]. Finally, publication bias was checked using the trim and fill procedure; we used Rosenthal’s estimator and the fail-safe number to analyze publication bias [22]. Finally, meta-regression analyses were utilized for the following variables: article publication year, gender, age, and dose. Regression models were applied for continuous variables. Meta-regressions were performed when the number of studies containing the variables to be analyzed was ≥4. PROMETA 3 software (IDo Statistics-Internovi, Cesena, Italy) was used. The considered outcomes were body mass index (BMI), homeostasis model assessment-insulin resistance index (HOMA-IR), glycosylated hemoglobin (Hb1Ac), Triglycerides (TG), Total Cholesterol (TC), High-density lipoprotein (HDL), and LDL. All of the values were reported in mg/mL while using a conversion formula [23].

## 4. Results

The literature search highlighted the presence of 529 references (Figure 1). After removing the duplicates, 358 papers were screened. Twenty-three full texts were verified. 16 were excluded and seven were included in the meta-analysis.

Table 1 reports the characteristics of the primary studies and results of outcomes. Table 2 shows the results of meta-analysis. We highlight that the papers included in this meta-analysis showed a low risk of bias: Supplementary Table S2 reports the results of risk bias assessment. The results of meta-regressions are showed in Table S3. Figures S1–S7 show results of sensitivity analysis.

### 4.1. BMI

**Figure 2:**
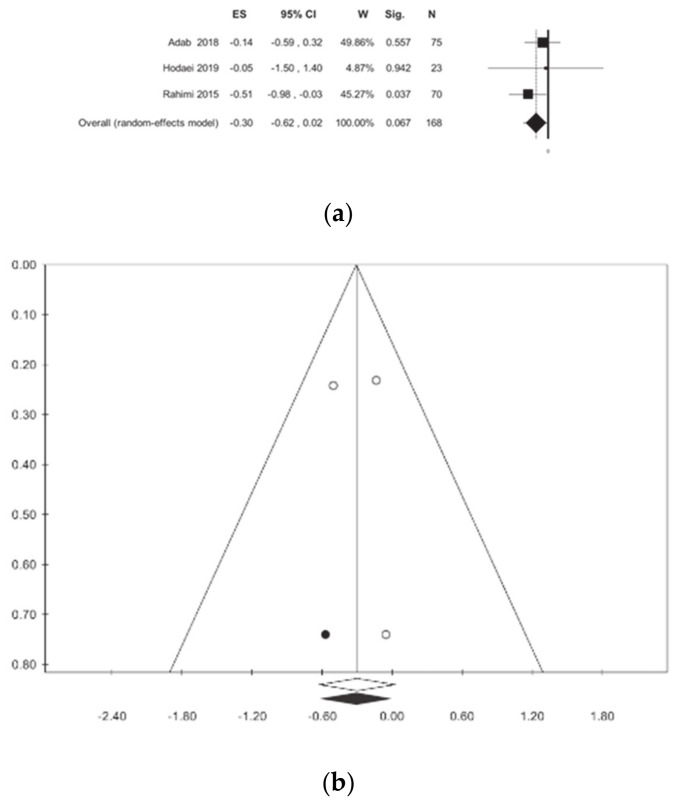
Meta-analysis results according to body mass index (BMI): (**a**) forest plot (**b**) funnel plot.

BMI was investigated in three studies [10,12,13] involving a total of 168 patients. Overall, curcumin treatment does not show a statistically significant reduction between the treated and untreated patients: this results in the absence of statistical heterogeneity (Table 2, Figure 2).

### 4.2. Hb1Ac

**Figure 3:**
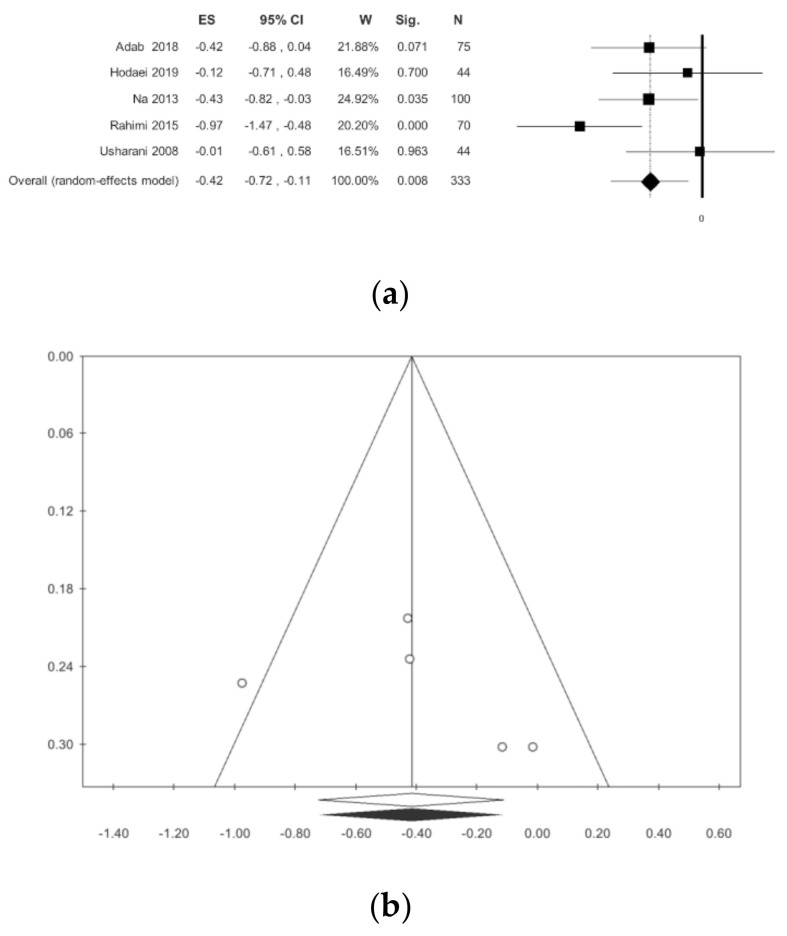
Meta-analysis results according to glycosylated hemoglobin (Hb1Ac): (**a**) forest plot (**b**) funnel plot.

Glycosylated hemoglobin was evaluated in five studies [10,12,13,15,16]. A statistically significant reduction was found in subjects that were treated with curcumin: −0.42 (−0.77; −0.11) *p* = 0.008, with moderate heterogeneity (I^2^ = 42.42), but not statistically significant (*p* = 0.107). Publication bias analysis did not highlight any differences between the observed and estimated values. It should be emphasized that there is a difference between the studies conducted in Iran and those conducted in other countries (Table 2, Figure 3); however, this difference is not statistically significant (ANOVA Q test *p* = 0.443). The meta-regressions only showed statistical significance for gender (*p* = 0.034).

### 4.3. HOMA

**Figure 4:**
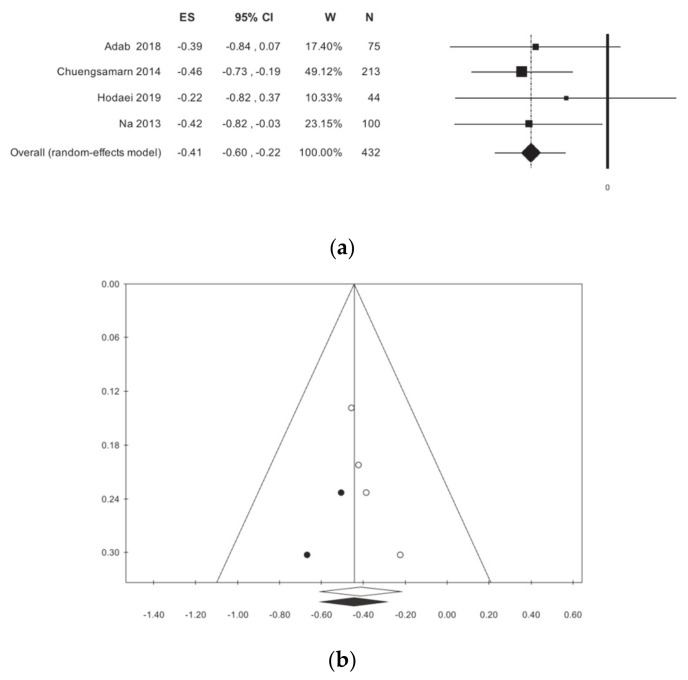
Meta-analysis results according to homeostasis model assessment (HOMA): (**a**) forest plot (**b**) funnel plot.

HOMA was detected in four studies [10,12,14,15] for a total of 432 patients. There is a statistically significant reduction of this index, without statistical heterogeneity: −0.42 (−0.77; −0.11) *p* < 0.001, (Table 2, Figure 4). Publication bias analysis highlighted a difference between the observed and estimated values: 0.45 (−0.61; −0.28) *p* < 0.001, with two trimmed studies. The subgroup analysis showed a difference between the studies that were conducted in Iran and those conducted in other countries, however this difference is not statistically significant (Table 2). The meta-regressions, concerning the selected moderators, did not show any statistical significance (Table S3).

### 4.4. HDL

**Figure 5:**
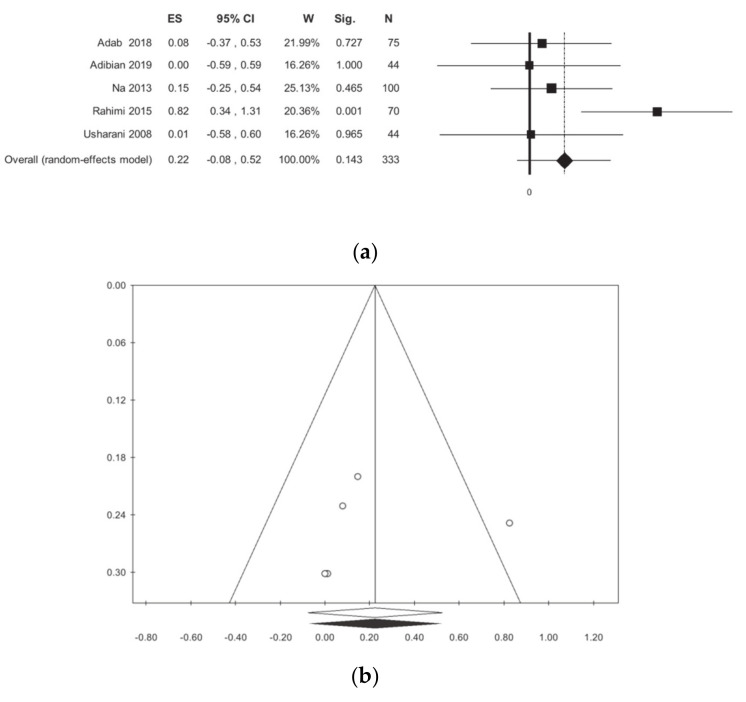
Meta-analysis results according to high-density lipoprotein (HDL): (**a**) forest plot (**b**) funnel plot.

HDL was evaluated in five studies for a total of 333 patients [11,12,13,15,16]. The analysis did not show statistically significant differences (Table 2, Figure 5). The subgroup analysis did not show a difference between the studies that were conducted in Iran and those conducted in other countries. Publication bias analysis did not highlight any differences between the observed and estimated values. Meta-regressions, regarding the selected moderators, did not show any statistical significance, with the exception of gender (*p* = 0.002) (Table S3).

### 4.5. LDL

**Figure 6:**
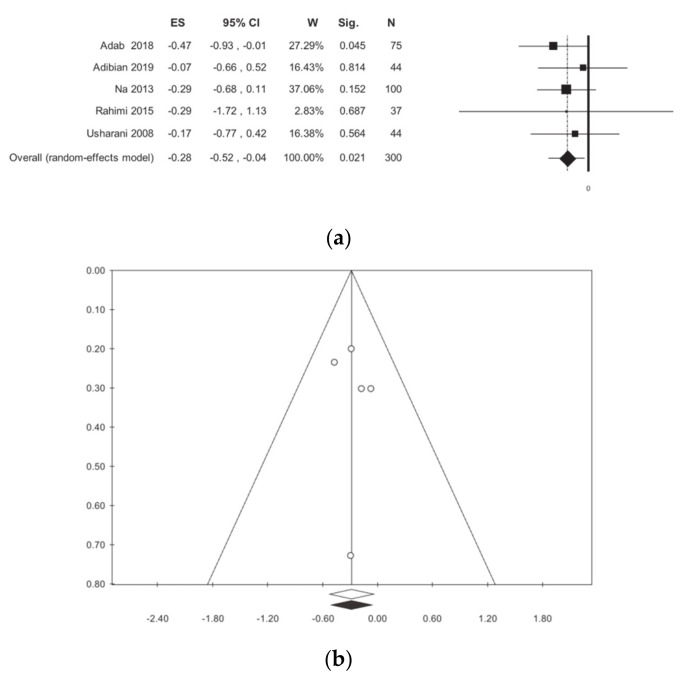
Meta-analysis results according to low-density lipoprotein (LDL): (**a**) forest plot (**b**) funnel plot.

The LDL dosage was evaluated in 300 patients for a total of five studies [11,12,13,15,16]. The meta-analysis showed a statistically significant reduction in curcumin-treated patients when compared to the placebo, with no statistical heterogeneity: −0.28 (−0.52; −0.04) *p* = 0.021, I^2^ 0.00 (Table 2, Figure 6). The subgroup analysis, as shown in Table 2, highlights a difference between studies that were conducted in Iran and those conducted outside Iran, but there is not a statistically significant difference. Publication bias analysis did not highlight any differences between the observed and estimated values. The meta-regressions, for the selected moderators, did not show any statistical significance (Table S3).

### 4.6. Triglycerides

**Figure 7:**
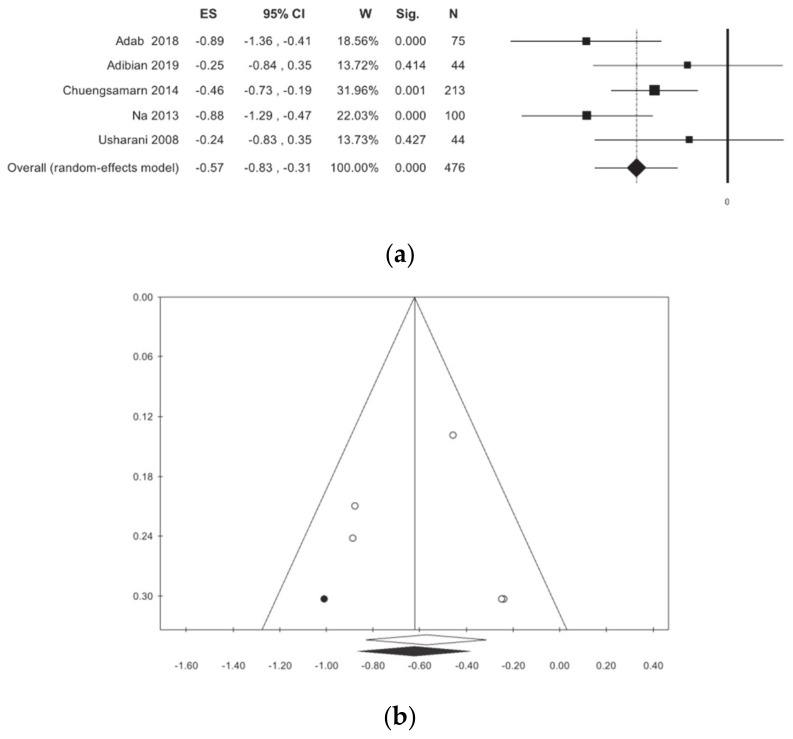
Meta-analysis results according to Triglycerides: (**a**) forest plot (**b**) funnel plot.

Triglycerides were evaluated in five primary studies, involving a total of 476 patients [11,12,13,14,15,16]. In patients treated with curcumin, a non-significant reduction in plasma triglyceride concentrations, without statistical heterogeneity, was identified (Table 2, Figure 7). The publication bias analysis highlighted a difference between observed and estimated values, respectively: −0.62 (−0.87; −0.37) (*p* < 0.001), −0.57 (−0.83; −0.31), with 1 trimmed study. The subgroup analysis revealed a non-statistically significant difference.

### 4.7. Total Cholesterol

**Figure 8:**
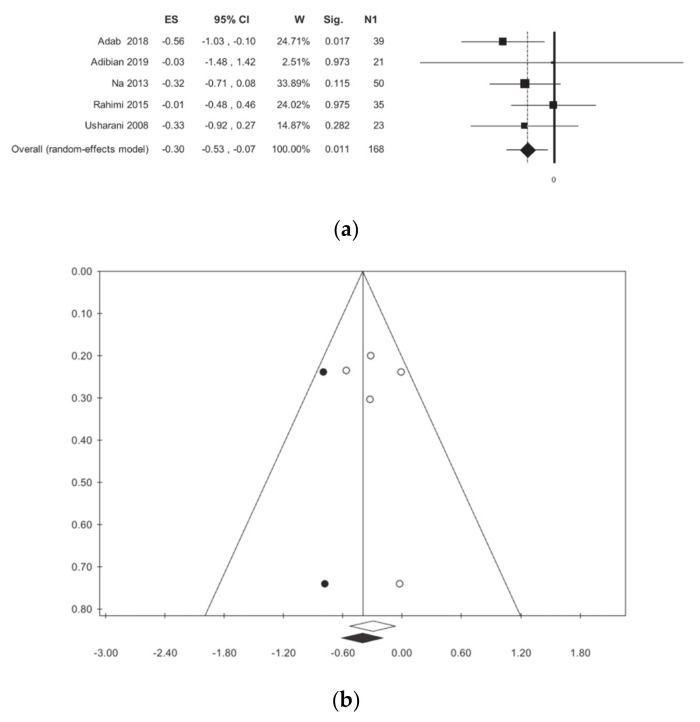
Meta-analysis results according to Total Cholesterol: (**a**) forest plot (**b**) funnel plot.

The total cholesterol was investigated in five studies for a total of 312 statistical units [11,12,13,15,16]. There is a reduction in cholesterol in curcumin-treated patients as compared to the placebo-treated patients, with no statistical heterogeneity (Table 2, Figure 8). Publication bias analysis highlighted a difference between the observed and estimated values, respectively: −0.30 (−0.53; −0.07) *p* < 0.001, −0.40 (−0.62; −0.28) (*p* < 0.001) with two trimmed studies. No statistically significant associations were found in meta-regressions (Table S3).

## 5. Discussion

In recent decades, there has been considerable interest among researchers in nutraceuticals and in particular, in naturally derived products, also known as natural health products (NHP), for the prevention, cure, and treatment of cardiovascular and metabolic diseases [24,25,26]. Some research indicates that chronically ill people tend to consume more NHP, and some surveys confirm that patients with T2DM are not excluded from this [27,28,29,30,31]. The pandemic spread of non-communicable diseases (NCD), and T2DM in particular, makes this population a target market of considerable interest for producers. The use of NHP in diabetics is linked to the co-treatment of hyperglycemia, dyslipidemia, and complications of diabetes. Among the various products, there is also curcumin. Currently, the FDA and EFSA recommend doses of curcumin of maximum 3 mg/kg/day, including the onset of toxicity for higher quantities (in particular, teratogenic effects, astrocytic cell abnormalities are reported, and it is not recommended in gallbladder stones) [5]. Overall, curcumin has a low bioavailability due to its hydrophobicity, so the pharmacologically active form is administered with a lipid vehicle or in association with piperine [5,32]. Specifically, for diabetes, curcumin also has an effect on hepatic lipogenesis, blocking the activity of the sterol regulatory element-binding protein gene (SREBP1) [6,33] and simultaneously activating the enzymes carnitine palmitoyltransferase 1 (CPT1) and acyl-CoA cholesterol acyltransferase (ACAT) that are involved in lipid mobilization [6,33].

The results of our meta-analysis seem to confirm this modulating capacity on lipid metabolism. The trials considered highlighted an overall reduction in LDL, TG, and TC in patients with uncomplicated T2DM. This result does not seem to be affected by statistical heterogeneity. There is a moderate publication bias. The low fail-safe for LDL, HDL, and TC indicates caution in the interpretation of the overall result, even if a high Rosenthal value allows for the observation found to be considered valid [22]. In order to confirm this, the subgroup analysis shows some differences that could be explained by the following considerations: method of conducting the study, quantity of curcumin administered, execution techniques, analysis, and collection of the blood chemistry method. The lack of influence of curcumin on HDL can be motivated by the fact that, notoriously, the increase in HDL is due globally to a more active lifestyle [34]. Similarly, the non-influence on BMI could be interpreted [35].

Because hypoglycemic properties of curcumin have been known since 1972 [36,37], the action is probably mediated by the inhibition of Phosporilase Kinase, which is to say, avoiding the mobilization of glucose from glycogen reserves [32,37,38,39]. Furthermore, curcumin would have a role in reducing the accumulation of advanced glycation end products [40] and the accumulation of these same metabolites at the level of the pancreatic insulae [41]. The inhibition of this process would mediate the Peroxisome Proliferator Activated Receptor Gamma (PPAR-Y), which, by increasing the amount of glutathione, would prevent oxidative damage that is caused by the state of hyperglycemia [42].

Hb1Ac and HOMA show significant results without statistical heterogeneity and publication bias. Regarding Hb1Ac, the subgroup analysis shows a difference between the studies that were conducted in Iran and those conducted outside Iran; on the contrary, this is not the case for HOMA, which shows concordant results. The hypoglycemic capacity of curcumin has also been tested in prediabetic populations with some success [43].

Concerning Hb1Ac and HDL, the results of the meta-regressions only show statistical significance with respect to gender.

## 6. Conclusions

Our meta-analysis seems to confirm the benefits on glucose metabolism, with results that appear to be more solid than those of lipid metabolism. In conclusion, the daily supplement of curcumin could improve some metabolic aspects of uncomplicated T2DM patients.

However, further studies are needed in order to test the efficacy and safety of curcumin in uncomplicated T2DM. The limitations of the present work can be attributed to some biases present in the primary studies: the small numbers of enrolled patients and the possible impact of grey literature. These aspects cannot be totally corrected through the meta-analytic technique.

## Figures and Tables

**Table 1:** The characteristics of included studies, according to intervention group (curcumins) and control groups (placebo) and results for each selected outcome.

Author, Year, Country	Mean Age	Male %	Diabetes Duration (Years)	Groups	BMI Variation Mean (SD)	HOMA-IR	Hb1Ac Mean (SD)	TG Mean (SD)	TC Mean (SD)	HDL Mean (SD)	LDL Mean (SD)
Hodaei 2019 Iran [10]	59	50	1–10	Intervention Group *N* = 21 (1500 mg)	29.2 (3.76) 28.9 (3.73)	62 (63) 62.4 (42)	11.3 (1.6) 11 (2.0)	-	-	-	-
				Control Group *N =* 23	28.2 (2.5) 28.1 (2.5)	53 (40) 65 (44)	11.2 (1.3) 11.1 (1.8)	-	-	-	-
Adibian 2019 Iran [11]	59	50	1–10	Intervention Group *N =* 21 (1500 mg)	-	-	-	124 (36) 109 (36)	167 (34) 163 (39)	30 (2) 30 (2)	112 (31) 108 (36)
				Control Group *N =* 23	-	-	-	126 (52) 121 (44)	180 (47) 175 (47)	30 (2) 30 (2)	125 (44) 118 (47)
Adab 2018 Iran [12]	55	51	5–10	Intervention Group *N =* 39 (2100 mg)	28.98 (3.68) 28.26. (3.45)	2.42 (1.73) 2.21 (1.43)	7.06 (1.01) 7.04 (0.98)	181.56 (79.9) 141.74 (52.02)	148.85 (36.11) 149.82 (35.67)	38.79 (10.30) 37.07 (9.12)	82.56 (20.99) 75.23 (18.84)
				Control Group N= 36	28.82 (4.96) 28.68 (4.86)	2.24 (1.48) 2.69 (2.02)	6.79 (1.08) 7.28 (1.59)	164.05 (81.19) 197.05 (96.98)	155.36 (36.27) 176.88 (37.58)	44.63 (10.66) 42.11 (9.39)	86.61 (21.99) 89.05 (21.46)
Rahimi 2015 Iran [13]	58.64	45	NR	Intervention Group (80 mg) *N =* 35	26.92 (2.71) 25.57 (2.71)	-	7.59 (1.74) 7.31 (1.54)	109 (94.75) 131 (60.27)	163.4 (33.94) 158.62 (44.06)	54.30 (14.02) 60.95 (15.68)	96.57 (33.94) 91.04 (28.72)
				Control Group *N =* 35	27.27 (3.59) 27.50 (3.38)	-	7.49 (1.75) 9.05 (2.33)	142 (97.5) 113 (58)	85.5 (15.3) 80.5 (9.1)	60.35 (15.96) 55.00 (11.09)	98.78 (30.33) 99.78 (30.33) 84.00 (12.59)
Chuengsamarn Thailand 2014 [14]	59	48	12	Intervention Group *N =* 107	-	9.58 (4.3) 4,32 (1.8)	-	219.12 (97.52) 141.99 (67.79)	-	-	-
				Control Groups *N =* 106	-	6.89 (2.67) 6.78 (2.5)	-	252.72 (114.12) 252 (114.12)	-	-	-
Na China 2013 [15]	55.07	49	7.6	Intervention Group (300 Mg) *N =* 50	-	5.80 (3.35) 4.14 (1.81)	7.77 (1.82) 7.02 (2.04)	223.8 (46.8) 157.5 (49.5)	540 (100) 493 (41.7)	52.9 (10.04) 54.8 (11.2)	166.0 (46.3) 146.7 (39.7)
				Control Group N= 50	-	5.82 (3.90) 5.49 (2.15)	7.72 (2.12) 7.99 (2.86)	193.8 (92.04) 186.7 (66.3)	538 (109) 522 (105.3)	51.35 (10.81) 51.74 (8.8)	166.8 (44.4) 160.2 (45.17)
Usharani India 2008 [16]		52	8	Intervention Group *N =* 23 (300 mg)	-	-	8.04 (0.85) 8.03 (0.76)	176.39 (27.61) 165.26 (25.78)	195.0 (41.16) 185.34 (34.35)	38.78 (7.69) 39.91 (0.68)	120.35 (42.13) 111.34 (37.65)
				Control Group N= 21	-	-	7.82 (0.57) 7.80 (0.62)	170.14 (47.54) 168.14 (47.10)	195.95 (35.72) 198.76 (35.09)	36.38 (7.67) 37.04 (5.92)	124.59 (34.94) 122.18 (35.56)

%: Males enrolled in each study.TG: triglycerides, TC: total cholesterol.

**Table 2:** Meta-Analysis results and moderator analysis (country: Iran versus outside Iran).

Outcome	K	Total Sample Size	Effect Size		Heterogeneity				Publication Bias			
			(95% CI)	*p*	I^2^	*p*	T^2^	T	Egger (*p*)	BEGGS (*p*)	Fail Safe (*n*)	Rosenthal (*n*)
BMI	3	168	−0.30 (−0.62, 0.02)	0.067	0.00	0.514	0.00	0.00	0.842	0.602	0	25
Hb1Ac	5	333	−0.42 (−0.77, −0.11)	0.008	42.42	0.107	0.06	0.24	0.501	0.327	12	35
Iran	3	189	−0.52 (−1.00, −0.04)	0.032	61.35	0.075	0.11	0.33				
Outside Iran	2	144	−0.28 (−0.67, 0.10)	0.153	22.22	0.257	0.02	0.14				
	ANOVA Q TEST *p* = 0.443											
HOMA	4	432	−0.41 (−0.66, −0.22)	<0.001	0.00	0.916	0.00	0.00	0.073	0.042	12	30
Iran	2	119	−0.33 (−0.69, 0.04)	0.078	0.00	0.667	0.00	0.00				
Outside Iran	2	313	−0.45 (−0.67, −0.22)	<0.001	0.00	0.885	0.00	0.00				
	ANOVA Q TEST *p* = 0.580											
HDL	5	333	0.22 (−0.08, 0.52)	0.143	45.91	0.116	0.05	0.27	0.856	0.327	1	35
Iran	3	189	0.31 (−0.21, 0.839	0.241	68.28	0.043	0.22	0.41				
Outside Iran	2	145	0.11 (−0.22, 0.43)	0.527	0.00	0.713	0.00	0.00				
	ANOVA Q TEST: *p* = 0.512											
LDL	5	300	−0.28 (−0.52, −0.04)	0.021	0.00	0.083	0.00	0.00	0.646	0.624	1	35
Iran	3	156	−0.32 (−0.67, 0.03)	0.077	0.00	0.582	0.00	0.00				
Outside Iran	2	144	−0.25 (−0.77, 0.42)	0.130	0.00	0.754	0.00	0.00				
	ANOVA Q TEST: *p* = 0.793											
TG	5	476	−0.57 (−0.83, −0.31)	<0.001	41.56	0.144	0.04	0.19	0.943	0.322	37	35
Iran	2	119	−0.59 (−1.22, 0.03)	0.063	63.77	0.099	0.13	0.36				
Outside Iran	3	357	−0.55 (−0.88, −0.22)	<0.001	49.19	0.140	0.04	0.20				
	ANOVA Q TEST *p* = 0.904											
TC	5	312	−0.30 (−0.53, −0.07)	0.01	0.00	0.573	0.00	0.00	0.975	1.00	3	30
Iran	3	168	−0.27 (−0.69, 0.15)	0.211	30.47	0.237	0.10	0.31				
Outside Iran	2	144	−0.32 (−0.65, 0.01)	0.056	0.00	0.979	0.00	0.00				
	ANOVA Q TEST *p* = 0.847											

## Data Availability

Not applicable.

## Supplementary Materials

The following are available online at https://www.mdpi.com/2072-6643/13/2/404/s1, Table S1: Prisma Checklist, Table S2: Risk of bias assessment, Table S3: Meta-regressions Results, Figures S1–S7: Sensitivity Analysis.

## Founding

The founding sponsors had no role in the design of the study; in the collection, analyses, or interpretation of data; in the writing of the manuscript, and in the decision to publish the results.
